# The Prevalence and Molecular Spectrum of α- and β-Globin Gene Mutations in 14,332 Families of Guangdong Province, China

**DOI:** 10.1371/journal.pone.0089855

**Published:** 2014-02-27

**Authors:** Aihua Yin, Bing Li, Mingyong Luo, Longchang Xu, Li Wu, Liang Zhang, Yuanzhu Ma, Tingting Chen, Shuang Gao, Juqing Liang, Hao Guo, Danqing Qin, Jicheng Wang, Tenglong Yuan, Yixia Wang, Wei-wei Huang, Wen-Fei He, Yanxia Zhang, Chang Liu, Sujian Xia, Qingshan Chen, Qingguo Zhao, Xiaozhuang Zhang

**Affiliations:** 1 Prenatal Diagnosis Centre, Guangdong Women and Children Hospital, Guangzhou, Guangdong, China; 2 Maternal and Children Metabolic-Genetic Key Laboratory, Guangdong Women and Children Hospital, Guangzhou, Guangdong, China; 3 Thalassemia Diagnosis Centre, Guangdong Women and Children Hospital, Guangzhou, Guangdong, China; 4 Department of Healthcare, Guangdong Women And Children Hospital, Guangzhou, Guangdong, China; 5 BioChain (Beijing) Science and Technology Inc., Beijing, China; 6 Department of Epidemiology, Medical College, Jinan University, Guangzhou, Guangdong, China; 7 Department of Health Statistics, Medical College, Jinan University, Guangzhou, Guangdong, China; Odense University hospital, Denmark

## Abstract

**Objective:**

To reveal the familial prevalence and molecular variation of α- and β-globin gene mutations in Guangdong Province.

**Methods:**

A total of 40,808 blood samples from 14,332 families were obtained and analyzed for both hematological and molecular parameters.

**Results:**

A high prevalence of α- and β-globin gene mutations was found. Overall, 17.70% of pregnant women, 15.94% of their husbands, 16.03% of neonates, and 16.83% of couples (pregnant women and their husbands) were heterozygous carriers of α- or β-thalassemia. The regions with the highest prevalence were the mountainous and western regions, followed by the Pearl River Delta; the region with the lowest prevalence was Chaoshan. The total familial carrier rate (both spouses were α- or β-thalassemia carriers) was 1.87%, and the individual carrier rates of α- and β-thalassemia were 1.68% and 0.20%, respectively. The total rate of moderate-to-severe fetal thalassemia was 12.78% among couples in which both parents were carriers.

**Conclusions:**

There was a high prevalence of α- and β-thalassemia in Guangdong Province. This study will contribute to the development of thalassemia prevention and control strategies in Guangdong Province.

## Introduction

Thalassemia is an autosomal recessive heritable blood disorder resulting from hemoglobin-production deficiency [1], [2]. It is one of the most common monogenic disorders in the world and is mainly endemic in some areas of the tropics and subtropics, including southern China [3]. There are two types of thalassemia, α- and β-thalassemia. Most patients with severe α-thalassemia may die *in utero* or shortly after birth as a result of serious intrauterine anemia, and most patients with severe β-thalassemia may develop serious anemia in early childhood if untreated. Thalassemia is an important public health problem in many countries, and its prevention is mainly dependent on prenatal diagnosis and genetic counseling.

In China, thalassemia is widely distributed on the southern bank of the Yangtze River [4], particularly in southern China, in the Guangdong, Guangxi and Hainan Provinces [5], [6], [7], [8], [9]. Previous studies have reported an estimated carrier rate of 3.16–11.72% for α-thalassemia and 1.96–3.87% for β-thalassemia in some regions of Guangdong Province [9], [10]; however, these studies may not reveal the true prevalence of thalassemia in Guangdong province because of a limited sampling area and sample size. Furthermore, the main aim of a thalassemia prevention and control program is to prevent the birth of infants with moderate-to-severe thalassemia, so pregnant women and their husbands are critical targets of such programs. Pregnant women, their fetuses and their husbands were enrolled in the study, and a large-scale familial investigation was conducted in 21 regions of Guangdong Province to reveal the familial prevalence of thalassemia and a provide scientific basis for thalassemia prevention and control in the province.

## Materials and Methods

### Study design and subjects

A two-stage cluster-sampling method was employed in the study, and the sampling area covered all 21 regions of Guangdong Province. In the first stage, we randomly sampled one county in each of the twenty-one regions of Guangdong Province. In the second stage, we sampled one or several hospitals with qualified midwives on staff in each county; in all, 91 hospitals were included in our study. Among 91 sampling hospitals with qualified midwives, 58.2% ((53/91)) of them are located in urban areas and 41.8% (38/91) are located in rural areas; and for grade of sampling hospitals, 2.2% (2/91), 13.2% (12/91), 42.9% (39/91) and 41.8% (38/91)of them are respectively provincial, municipal, county, town and community level (Table 1, Table S1). From each hospital, we selected pregnant women who were going to deliver between May and August 2012 and their husbands. The inclusion criteria were that one or both of the spouses were of Guangdong ancestry. After obtaining written informed consent from all subjects, we collected peripheral venous blood from the pregnant women and their husbands as well as umbilical blood samples. In total, 14,332 families were initially contacted to participate to this study. Among all the couples, the people who were not Guangdong ancestry and the unqualified samples were excluded for this study. After selected, 40,808 blood samples (13,386 pregnant women, 13,148 husbands and 14,274 umbilical blood samples) were included in the final statistical analysis.

**Table 1:** The situation of category of sampling hospitals with qualified midwives.

Category	The number of sampling hospitals with qualified midwives	Percentage (%)
Urban or rural		
urban	53	58.2
Rural	38	41.8
Grade of sampling hospitals		
Provincial level	2	2.2
Municipal level	12	13.2
County level	39	42.9
Town and community level	38	41.8

### Ethical declaration

The authors declare that the experiments comply with the current laws of China and gain informed consent of all the subjects before joining the study which had the approval by Medical Ethics Committee of Guangdong Women and Children Hospital.

### Hematological analysis

The blood samples were collected consecutively from 14,332 families between May and August 2012 in the sampled hospitals in twenty-one regions of Guangdong Province. The blood samples (3 ml) from all subjects were collected in EDTA tubes; routine blood tests were performed, and the samples were transported on ice to Guangdong Women and Children's Hospital for further analysis. Automatic capillary electrophoresis (Sebia, France) was used to assess the concentration of the hemoglobins A, A2 and F as well as any abnormal hemoglobin variants, including Hb Bart's, Hb Constant Spring and Hb J.

### Molecular analysis

Genomic DNA was extracted from all peripheral venous blood and umbilical blood samples using an automation system Lab-Aid 820 (Zee San Biotech Company, Fujian, China). Twenty-three mutations, including three deletions associated with α-thalassemia, three non-deletional mutations associated with α-thalassemia, and seventeen point mutations associated with β-thalassemia, were identified using a suspension-array system developed by our lab, the sensitivity and specificity of which has been verified for various types of gene mutation; this system has been patented in the People's Republic of China (Pub. No.: WO/2012/136070). The method is based on the Luminex xMAP system, which was successfully applied to the genotyping of human papillomavirus (HPV) [11]. The procedure involved probe design, multiplex PCR, the attachment of probes to microspheres, hybridization and analysis. A single operator can complete the entire procedure in five hours. This system can accurately diagnose the genotype associated with thalassemia with high throughput. The 23 mutations we tested were most common and high incidence in Southern China which has been validated by several researches[9], [11], [12], including deletional α-globin mutations (the Southeast-Asian deletion (–SEA), the rightward deletion (−α3.7) and the leftward deletion (−α4.2)), point mutations associated with α-thalassemia (Hb Constant Spring, Hb Quong Sze and Hb Westmead) and the seventeen point mutations associated with β-thalassemia (codon 41/42 (–TCTT), 654, −29 (A>G), −28 (A>G), codon 71/72 (+A), codon 17 (A>T), codon 43 (G>T), Hb E [β26(B8)Glu→Lys, GAG>AAG or codon 26 (G>A)], codon 27/28 (+C), codon 31 (–C), −32 (C>A), −30 (T>C), codon 14/15 (+G), IVS-I-1 (G>T), IVS-I-5 (G>T), Int and Cap). The results of the molecular analysis with the suspension-array system were verified using a Gap-PCR kit (Shenzhen Yaneng Bio) for deletion mutations associated with α-thalassemia and direct genomic sequencing for non-deletional mutations associated with α-thalassemia and point mutations associated with β-thalassemia.

### Statistical analysis

Statistical analysis was conducted using the SPSS software (Ver. 13, SPSS Inc., Chicago, USA). The prevalence of familial thalassemia was evaluated by descriptive statistics. Bootstrap method was used to estimate the sampling error for the prevalences of thalassemia mutations.

## Results

### The prevalences of α- and β-globin gene mutations among the pregnant women, their husbands and neonates

Among 13,386 pregnant women and 13,148 of their husbands of Guangdong ancestry, the total number of α- and β-globin gene mutations was 4,732 (17.83%); there were 3,531 α-globin gene mutations (13.31%), with mutation rates of 6.85% for the —^SEA^ deletion, 3.68% for the −α^3.7^ deletion, and 1.27% for the −α^4.2^ deletion; the remaining 1201 mutations were in the β-globin gene (4.53%), with mutation rates of 1.78% for the 41/42 (-CTTT) mutation and 1.18% for the IVS-II-654 (C→T) mutation. The prevalence of α- and β-globin gene mutations among the pregnant women, their husbands and the 14,274 neonates of Guangdong ancestry was similar proportionately to that observed in the total population of pregnant women and husbands of Guangdong ancestry (Table 2).

**Table 2:** The prevalences of α- and β-globin gene mutations among the pregnant women, their husbands and neonates in Guangdong Province, China.

Mutation	Pregnant women		Husbands		Neonates		Pregnant women + Husbands	
	n	%	n	%	n	%	n	%
**α-Thalassemia**								
—^SEA^	992±30	7.41±0.22	825±26	6.27±0.20	931±30	6.52±0.21	1817±40	6.85±0.15
-α^3.7^	501±21	3.74±0.16	475±22	3.61±0.17	487±22	3.41±0.15	976±31	3.68±0.12
-α^4.2^	192±14	1.43±0.10	145±12	1.1±0.09	177±13	1.24±0.09	337±18	1.27±0.07
-α^CS^	53±7	0.4±0.05	44±7	0.33±0.05	45±6	0.32±0.04	97±10	0.37±0.04
-α^QS^	30±5	0.22±0.04	22±5	0.17±0.04	25±5	0.18±0.04	52±7	0.2±0.03
-α^WS^	134±12	1±0.09	118±11	0.9±0.08	138±12	0.97±0.08	252±17	0.95±0.06
α-Thalassemia Total	1902±41	14.21±0.31	1629±37	12.39±0.28	1803±40	12.63±0.28	3531±56	13.31±0.21
**β-Thalassemia**								
Codons 41/42 (-CTTT)	243±15	1.82±0.11	228±15	1.73±0.11	258±16	1.81±0.11	471±21	1.78±0.08
IVS-II-654 (C→T)	171±13	1.28±0.10	141±12	1.07±0.09	156±12	1.09±0.08	312±17	1.18±0.06
-29 (A→G)	10±3	0.07±0.02	9±3	0.07±0.02	9±3	0.06±0.02	19±5	0.07±0.02
-28 (A→G)	95±9	0.71±0.07	75±8	0.57±0.06	86±10	0.6±0.07	170±13	0.64±0.05
Condons 71/72 (+A)	15±4	0.11±0.03	14±4	0.11±0.03	12±4	0.08±0.03	29±6	0.11±0.02
Condon 17, A>T	53±7	0.4±0.05	45±7	0.34±0.05	46±7	0.32±0.05	98±10	0.37±0.04
Condon 43 (G→T)	3±2	0.02±0.01	2±1	0.02±0.01	3±2	0.02±0.01	5±2	0.02±0.01
E	15±4	0.11±0.03	16±4	0.12±0.03	15±4	0.11±0.03	31±5	0.12±0.02
Codons 27/28 (+C)	9±3	0.07±0.02	10±3	0.08±0.02	11±3	0.08±0.02	19±4	0.07±0.02
14-15	6±2	0.04±0.01	4±2	0.03±0.02	2±1	0.01±0.01	10±3	0.04±0.01
IVS-I-1 (G→T)	4±2	0.03±0.01	4±2	0.03±0.02	7±3	0.05±0.02	8±3	0.03±0.01
IVS-II-5 (G→C)	2±1	0.01±0.01	1±1	0.01±0.01	1±1	0.01±0.01	3±2	0.01±0.01
Int	0±0	0±0.00	1±1	0.01±0.01	0	0±0.00	1±1	0±0.00
Cap	10±3	0.07±0.02	15±4	0.11±0.03	13±4	0.09±0.03	25±5	0.09±0.02
β-Thalassemia Total	636±25	4.75±0.19	565±23	4.3±0.17	619±23	4.34±0.16	1201±34	4.53±0.13
**Total**	2538±46	18.96±0.34	2194±42	16.69±0.32	2422±45	16.97±0.32	4732±63	17.83±0.24

In all, 4,725 deletion mutations associated with α-thalassemia were verified using the Gap-PCR kit; 609 non-deletional mutations associated with α-thalassemia and 1,820 point mutations associated with β-thalassemia were verified by direct genomic sequencing, and 341 samples randomly selected from 34,054 samples with negative results were also confirmed by corresponding above-mentioned methods.

### The rates of α- and β-thalassemia carrier status among the pregnant women, their husbands and neonates

Among the statistical samples, there were 4,465 thalassemia carriers (16.83%); of these, 3,268 (12.32%) were carriers of α-thalassemia alone, 1,027(3.87%) were carriers of β-thalassemia alone and 170 (0.64%) were carriers of both α- and β-thalassemia. The prevalence of the α- and β-thalassemia carrier status among the pregnant women and their husbands of Guangdong ancestry and the 14,274 neonates with one or both parents of Guangdong ancestry were proportionally similar to that observed in the total population of pregnant women and husbands of Guangdong ancestry (Table 3).

**Table 3:** The rates of α- and β-thalassemia carrier status among pregnant women, their husbands and neonates in Guangdong Province, China.

Variable	Pregnant women		Husbands		Neonates		Pregnant women+ Husbands	
	N	%	N	%	N	%	N	%
α-Thalassemia carrier only	1,735±38	12.96±0.28	1,533±38	11.66±0.29	1,675±38	11.73±0.27	3,268±54	12.32±0.20
β-Thalassemia carrier only	533±23	3.98±0.17	494±22	3.76±0.17	533±22	3.73±0.15	1,027±33	3.87±0.12
Both α and β-Thalassemia carrier	102±10	0.76±0.07	68±8	0.52±0.06	81±9	0.57±0.06	170±13	0.64±0.05
Total carrier	2,370±44	17.70±0.33	2,095±41	15.93±0.32	2,289±44	16.04±0.31	4,465±61	16.83±0.23

### The rates of α- and β-thalassemia carrier status among the pregnant women and their husbands in the 21 regions of Guangdong Province

**Figure 1:**
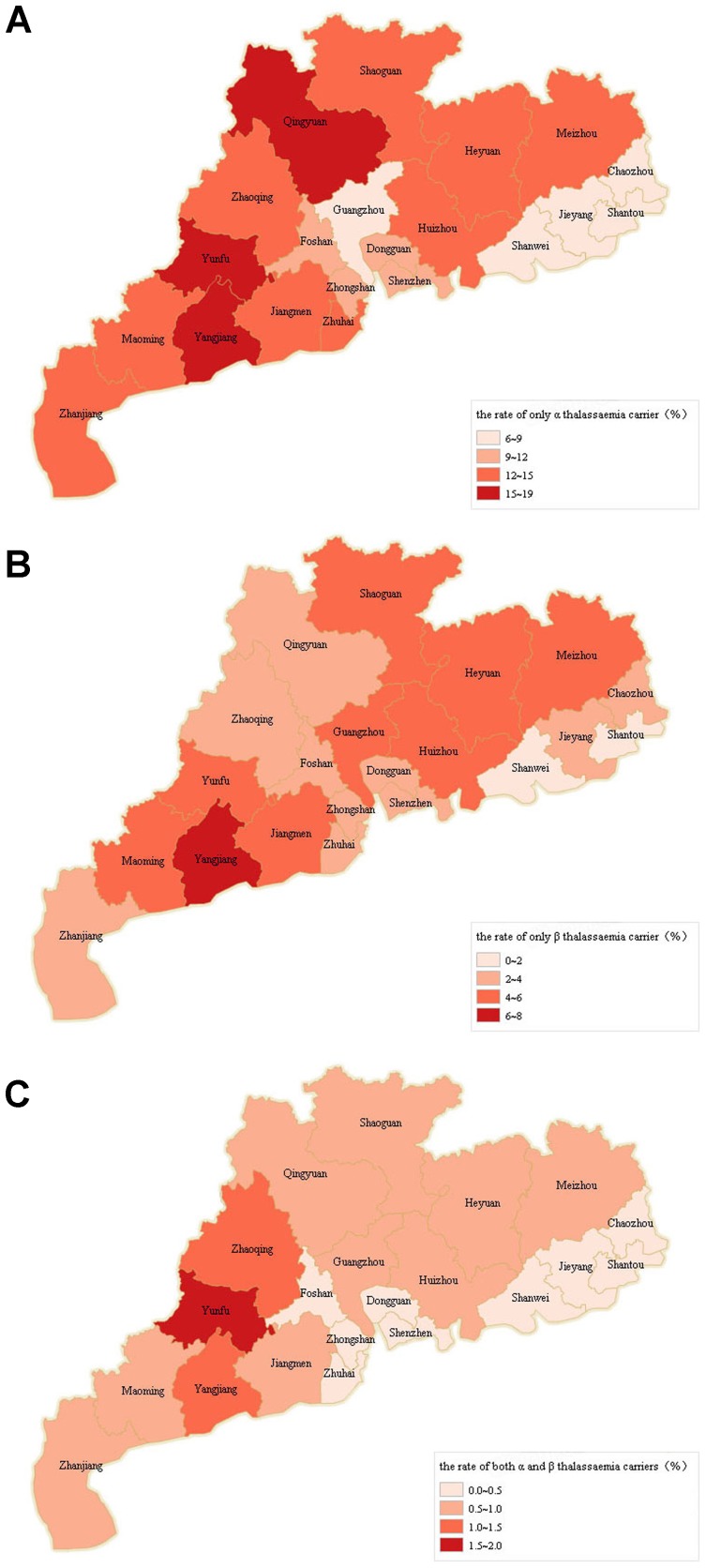
The rates of carrier status among pregnant women and their husbands in the 21 regions of Guangdong Province, China. A) α-thalassemia only; B) β-thalassemia; C) α- and β-thalassemia.

Among the 21 regions of Guangdong Province, the rate of α-thalassemia carrier status in the 13386 pregnant women (ancestry data were missing for 799 subjects) and 13,148 husbands (ancestry data were missing for 1195 subjects) of Guangdong ancestry varied between 6.03 and 18.13. The rate is higher in mountainous regions (including Yunfu, Qingyuan, Meizhou, Heyuan and Shaoguan) and in western regions (including Yangjiang, Maoming and Zhanjiang) and is lowest in Chaoshan (including Jieyang, Chaozhou, Shanwei and Shantou; Fig. 1A).

For β-thalassemia carrier, the rate varied between 1.31 and 6.02, which is higher in the mountainous regions and western regions and is lowest in Chaoshan (Fig. 1B).

The rate of α- and β-thalassemia carrier showed less variation, ranging from 0.15 to 1.89. The distributed status was similar to that of β-thalassemia carrier (Fig. 1C).

### Distributions of the α- and β-globin genotypes and the frequencies of α- and β-thalassemia

Among the 13,386 pregnant women of Guangdong ancestry, 1,837 were carriers of α-thalassemia, and —SEA/αα was the most common mutation, accounting for more half of all α-thalassemia genotypes (51.71%). Other high-prevalence genotypes were -α3.7/αα, -α4.2/αα or αWSα/αα. Overall, these four genotypes accounted for 92.43% of all α-thalassemia genotypes. The rates of carrier status among the 13,148 husbands of Guangdong ancestry and 14,274 neonates with one or both parents of Guangdong ancestry were 94.56% and 93.68%, respectively (Table 4).

**Table 4:** α-globin genotypes and α-thalassemia frequencies among pregnant women, their husbands and neonates in Guangdong Province, China.

Genotype	Pregnant women		Husbands		Neonates	
	n	%	n	%	n	%
—SEA/—SEA	—	—	—	—	5	0.28
—SEA/-α3.7	23	1.25	12	0.75	17	0.97
—SEA/-α4.2	10	0.54	4	0.25	6	0.34
—SEA/αCSα	3	0.16	2	0.12	1	0.06
—SEA/αQSα	1	0.05	1	0.06	1	0.06
—SEA/αWSα	5	0.27	0	0.00	1	0.06
—SEA/αα	950	51.71	806	50.34	895	50.97
-α3.7/-α3.7	6	0.33	2	0.12	3	0.17
-α3.7/-α4.2	7	0.38	0	0.00	7	0.40
-α3.7/αCSα	2	0.11	2	0.12	1	0.06
-α3.7/αQSα	2	0.11	1	0.06	0	0.00
-α3.7/αWSα	3	0.16	2	0.12	2	0.11
-α3.7/αα	452	24.61	454	28.36	454	25.85
-α4.2/αCSα	0	0.00	1	0.06	1	0.06
-α4.2/αQSα	1	0.05	0	0.00	2	0.11
-α4.2/αWSα	2	0.11	1	0.06	0	0.00
-α4.2/αα	172	9.36	139	8.68	161	9.17
αCSα/αα	48	2.61	39	2.44	42	2.39
αQSα/αα	26	1.42	20	1.25	22	1.25
αWSα/αα	124	6.75	115	7.18	135	7.69
Total	1837	100.00	1601	100.00	1756	100.00

The results displayed that635 pregnant women were carriers of β-thalassemia, and β41-42/βA was the most common mutation, accounting for almost 40% of all β-thalassemia genotypes (38.27%). Most of the remaining genotypes were β654/βA, β-28/βA or β17/βA. Overall, these four genotypes accounted for 88.19% of all β-thalassemia genotypes. The rates of carrier status among the husbands and the 14,274 neonates were 86.48% and 87.47%, respectively (Table 5).

**Table 5:** β-globin genotypes and β-thalassemia frequencies among pregnant women, their husbands and neonates in Guangdong Province, China.

Genotype	Pregnant women		Husbands		Neonates	
	n	%	n	%	n	%
β-28/β-28	0	0.00	0	0.00	1	0.16
β-28/β17	1	0.16	0	0.00	0	0.00
β-28/βA	94	14.80	74	13.17	82	13.36
β-28/βE	0	0.00	1	0.18	0	0.00
β-29/βA	10	1.57	9	1.60	9	1.47
β14-15/βA	6	0.94	4	0.71	2	0.33
β17/β41-42	0	0.00	0	0.00	1	0.16
β17/βA	52	8.19	45	8.01	45	7.33
β27-28/βA	9	1.42	10	1.78	11	1.79
β41-42/β-28	0	0.00	0	0.00	2	0.33
β41-42/βA	243	38.27	226	40.21	254	41.37
β41-42/βCap	0	0.00	1	0.18	1	0.16
β43/βA	3	0.47	2	0.36	3	0.49
β654/βA	171	26.93	141	25.09	156	25.41
β71-72/βA	15	2.36	14	2.49	12	1.95
βCap/βA	10	1.57	14	2.49	12	1.95
βE/β41-42	0	0.00	1	0.18	0	0.00
βE/βA	15	2.36	14	2.49	15	2.44
βInt/βA	0	0.00	1	0.18	0	0.00
βIVS-1/βA	4	0.63	4	0.71	7	1.14
βIVS-5/βA	2	0.31	1	0.18	1	0.16
Total	635	100.00	562	100.00	614	100.00

### The frequencies of carrying genes for the same type of thalassemia among couples

Thalassemia is one of the commonest autosomal recessive hemoglobin disorders; the couples carry the same type of thalassemia has a high risk to have a moderate to severe thalassemia fetus. The main approach of thalassemia prevention and control is to prevent birth of these moderate to severe thalassemia fetus. Therefore, we derived the “familial carrying rate”, i.e., the rate at which couples carry genes for the same type of thalassemia. In total, 266 of the 14,332 couples included two carriers of the same thalassemia genotype (genotype data were missing for 132 individuals). The total familial carrying rate was 1.87%, and the familial carrying rates of α- and β-thalassemia were 1.68% and 0.20%, respectively (Table 6).

**Table 6:** The frequenciess of carrying genes for the same type of thalassemia among couples in Guangdong Province, China.

Variable	The number of couple (N)	Family-based carrying rate (%)
Both α-carriers	238	1.68
Both β-carriers	28	0.20
Both α and β-carriers	0	0
Total	266	1.87

### The probabilities of moderate-to-severe fetal thalassemia

The standard strategy of laboratory diagnosis used for moderate-to-severe fetal thalassemia was combined by phenotypic screening and genotyping. The screening for α- and β- thalassemia was carried out when the mean corpuscular volume (MCV) was <82fL and/or mean corpuscular Hb (MCH) was <27pg which indicate hypochromic microcytic anemia. Meanwhile, the serum iron and ferritin were measured for exclusion of iron deficiency anemia. In combination with the Hb A2 level that Hb A2<3.0% indicate α-thalassemia trait and Hb A2>3.5% indicate β- thalassemia trait. Then all such positive samples were further characterized by genotyping. Among the 266 couples carrying mutant genes for the same type of thalassemia, 34 had produced fetuses with moderate-to-severe thalassemia. The total rate moderate-to-severe fetal thalassemia was thus 12.78% (34/266) among the couples with the same type of thalassemia, and the rates of moderate-to-severe fetal α- and β-thalassemia were 12.61% (30/238) and 14.29% (4/28), respectively.

## Discussion

Previous studies have examined the prevalence and molecular spectrum of α- and β-globin gene mutations in Guangdong Province, but they were limited in sampling area and sample size; there is not a large-scale, large-sample and province-wide study conducted in Guangdong Province. Therefore, previous studies were of limited representative value and may not reveal the true prevalence of thalassemia in Guangdong Province. Our study had considerable financial support, and it has three key features. The first is the large scale, random sampling of one county in each of the twenty-one regions of Guangdong Province. The second is the family-based sampling; because the main aim of thalassemia intervention is to prevent the birth of infants with moderate-to-severe thalassemia, pregnancy is a critical period, and pregnant women and their husbands are critical subjects of intervention. Therefore, we selected pregnant women, their husbands and their fetuses as the subjects of our study. The third advantage is the large random sample. By scientific design and random sampling, we obtained a large random familial sample, including 14,332 families and 40,808 blood samples (13,386 peripheral venous blood samples from pregnant women, 13,148 peripheral venous blood samples from husbands, 14,274 umbilical blood samples). Therefore, our study could reveal the prevalence and molecular variation of α- and β-globin gene mutations in Guangdong Province.

We found a high prevalence of α- and β-globin gene mutations. Overall, the frequencies of α- and β-globin gene mutations are 18.96%, 16.69%, 16.97% and 17.83% among pregnant women, husbands, neonates and “pregnant women and husbands”, respectively. We also found a high prevalence of α- and β-thalassemia carrier status. The frequencies of carrier status for α-thalassemia alone were 12.96% of pregnant women, 11.66% of husbands, 11.73% of neonates, and 12.32% of pregnant women and husbands. The frequencies for β-thalassemia alone were 3.98% of pregnant women, 3.76% of husbands, 3.73% of neonates, and 3.87% of pregnant women and husbands. Finally, the frequencies for α- and β-thalassemia together were 0.76% of pregnant women, 0.52% of husbands, 0.57% of neonates, and 0.64% of pregnant women and husbands. Overall, 17.70% of pregnant women, 15.94% of husbands, 16.03% of neonates, and 16.83% of pregnant women and husbands in Guangdong Province were heterozygous carriers of α- and/or β-thalassemia. Comparing with other countries, the frequency of α-thalassemia reported in our study are lower than that reported in the north of Thailand and Laos (30%–40%) and higher than that reported in Malaysia (4.5%) and Filipine (5%)[13], and the frequency of β-thalassemia reported in our study are lower than that reported in Cyprus (14%)and Sardinia (10.3%) [14]. And comparing with previous studies in China, these rates are higher than those reported in previous studies in Guangdong Province and other provinces in southern China [9], [10], [15], [16], [17], [18], [19] but are lower than those reported in several studies in Guangxi, Yunnan and Guizhou Provinces [11], [20], [21], [22]. The potential reasons for these differences may include differences in the study population, sampling area and method of gene detection.

The prevalences of α- and β-thalassemia carrier status varied among the twenty-one regions of Guangdong Province. The regions with the highest prevalence were the mountainous region (including Yunfu, Qingyuan, Meizhou, Heyuan and Shaoguan) and the western region (including Yangjiang, Maoming and Zhanjiang), followed by the Pearl River Delta (including Guangzhou, Shenzhen, Foshan, Zhongshan, Dongguan, Zhuhai, Jiangmen, Zhaoqing and Huizhou). The lowest prevalence was found in Chaoshan (including Jieyang, Chaozhou, Shanwei and Shantou). The three regions with the highest prevalence of α-thalassemia carrier status were Yangjiang, Yunfu and Qingyuan; the three regions with the lowest prevalence were Shantou, Chaozhou and Shanwei. The three regions with the highest prevalence of β-thalassemia carrier status were Yunfu, Yangjiang and Meizhou; the three regions with the lowest prevalence were Shantou, Shanwei and Jieyang. In our study, the prevalence of β-thalassemia carrier status in Zhongshan City (2.70%) was slightly lower than that reported by Zhang CM in 2010 (3.07%) [23], and the results obtained for other cities were also higher than those reported in previous studies [12], [24], [25], [26], [27], [28], [29].

Among α-globin genotypes, the Southeast-Asian deletion (—SEA/αα) accounts for the greatest proportion in three populations (pregnant women: 51.71%, husbands: 50.34%, neonates: 50.97%), followed by –α3.7/αα (pregnant women: 24.61%, husbands: 28.36%, neonates: 25.85%) and –α4.2/αα (pregnant women: 9.36%, husbands: 8.68%, neonates: 9.17%). The study indicates that the Southeast-Asian deletion occurs most frequently; the above three genotypes account for nearly 90% of all α-globin genotypes in Guangdong Province. Among the β-globin genotypes, β41-42/βA accounts for the greatest proportion in the three populations (pregnant women: 38.27%, husbands: 40.21%, neonates: 41.37%), followed by β654/βA (pregnant women: 26.93%, husbands: 25.09%, neonates: 25.41%) and β-28/βA (pregnant women: 14.80%, husbands: 13.17%, neonates: 13.36%). The study indicates that β41-42/βA occurs most frequently; the above three genotypes account for nearly 80% of all β-globin genotypes in Guangdong Province. Comparing with other countries, the percentage of β-globin genotypes reported in our study are different from that reported in Vietnam, Thailand, India and SriLanka [30], [31], [32], [33]. And comparing with previous studies in China, the results are consistent with those reported by Xu XM in Guangdong Province [9] but differ from those reported by Zheng CG in Guangxi Province [5].

Because this study is family-based, we have coined the term “familial carrying rate”, i.e., the rate at which couples carry genes for the same type of thalassemia. This rate has not been described in previous studies. Our study reveals that total familial carrying rate is 1.87% among couples in which the pregnant woman and/or her husband are of Guangdong ancestry; the familial carrying rates of α- and β-thalassemia are 1.68% and 0.20%, respectively. Furthermore, our study also revealed that the total rate of moderate-to-severe fetal thalassemia is 12.78% among couples carrying genes for the same type of thalassemia; the rates of moderate-to-severe α- and β-thalassemia are 12.61% and 14.29%, respectively. We thus derive the probability of moderate-to-severe fetal thalassemia among the couples in which the pregnant woman and/or her husband are of Guangdong ancestry from the product of the above two rates (1.87%*12.78%; 0.24% in Guangdong Province in our study). According to the current annual birth rate in the population of Guangdong ancestry (approximately 1,300,000 in 2012), the estimated total incidence of moderate-to-severe fetal thalassemia would be almost three thousand cases (1,300,000*0.24%) every year in Guangdong Province. Furthermore, because some cases of induced labor may have been neglected in our study, the above number is likely an underestimate.

To our surprise, twenty-seven of the thirty-four cases of moderate-to-severe fetal thalassemia in our study resulted in live births, indicating that these twenty-seven families never received effectual thalassemia intervention, including prenatal screening, prenatal diagnosis and induced labor. Because of the high prevalence of thalassemia and low accessibility of thalassemia intervention, thalassemia remains a severe public health problem in Guangdong Province. The emphasis in thalassemia prevention and control should be placed on public health education, training doctors, establishing networks and the wide implementation of premarital and prenatal screening to increase the accessibility of thalassemia intervention and reduce (ultimately, to zero) the number of infants born with moderate-to-severe thalassemia. The government of Guangdong Province has committed to investing thirty-five million yuan for thalassemia prevention and control among pregnant women and their husbands every year. We also suggest the need for further research, especially on the factors influencing the accessibility of thalassemia intervention, to provide a scientific basis for government decision-making.

## Supporting Information

Table S1 **The situation of sampling.**
(XLS)
